# Transfer of Deoxynivalenol (DON) through Placenta, Colostrum and Milk from Sows to Their Offspring during Late Gestation and Lactation

**DOI:** 10.3390/toxins10120517

**Published:** 2018-12-04

**Authors:** Amin Sayyari, Silvio Uhlig, Christiane Kruse Fæste, Tore Framstad, Tore Sivertsen

**Affiliations:** 1 Department of Production Animal Clinical Sciences, Faculty of Veterinary Medicine, Norwegian University of Life Sciences, P.O. Box 369 Sentrum, 0102 Oslo, Norway; tore.framstad@nmbu.no (T.F.); tore.sivertsen@nmbu.no (T.S.); 2 Section for Chemistry, Norwegian Veterinary Institute, P.O. Box 750 Sentrum, 0106 Oslo, Norway; silvio.uhlig@vetinst.no; 3 Toxinology Research Group, Norwegian Veterinary Institute, P.O. Box 750 Sentrum, 0106 Oslo, Norway; christiane.faste@vetinst.no

**Keywords:** deoxynivalenol, placental transfer, lactational transfer, piglets, sows

## Abstract

Deoxynivalenol (DON) contamination of feed may result in reduced growth, feed refusal, immunosuppression, and health problems in swine. Piglets can be exposed to DON via placenta before birth and via milk during lactation. The extent of early-life exposure of piglets to DON is, however, not fully known. This study was therefore aimed at investigating DON uptake in sows fed with naturally contaminated diets, DON transfer across placenta during late gestation, and transfer of DON to piglets via colostrum and milk. Forty-four crossbred sows were evaluated from day 93 ± 1 of gestation until weaning of piglets and fed with feed made from naturally DON-contaminated oats at three concentration levels: (1) control (DON < 0.2 mg/kg), (2) DON level 1 (1.4 mg DON/kg), and (3) DON level 2 (1.7 mg DON/kg). The transfer of DON to the piglets was evaluated in 15 sows, with repeated sampling of blood and milk from the sows and blood samples from five piglets of each litter. The piglet/sow plasma DON ratio and milk/plasma (M/P) DON ratio in sows were calculated to estimate the degree of transfer. Piglet/sow plasma ratios were 2.14 at birth, 2.30 within 12–36 h after parturition, 0.08 on day 7, 0.16 on day 21, and 0.20 at weaning. M/P ratios were 0.92, 1.11, 0.94, 1.21, and 0.90, respectively. The results indicate that DON is efficiently transferred across placenta and into milk. However, the low piglet/sow plasma ratios at mid-lactation to weaning indicate that the piglets were most strongly exposed to DON in early life, despite the high M/P ratios and efficient secretion of DON in milk throughout the entire lactation.

## 1. Introduction

Mycotoxins are secondary metabolites of fungi that have a variety of adverse effects on animals and humans [1,2]. One of the most common mycotoxins produced by *Fusarium* fungi is the trichothecene deoxynivalenol (DON) [1]. DON is produced mainly by *F. graminearum* and *F. culmorum,* and is the most commonly detected trichothecene worldwide [3]. This mycotoxin can contaminate wheat, oats, maize, and barley, and is known to cause significant economic losses in farm animal production, particularly in pigs [4]. The following rank order of decreasing sensitivity: pigs > mice > rats > poultry ≈ ruminants, shows that susceptibility to DON differs between animal species [2,5]. This is, in part, related to differences in metabolism, absorption, distribution, and elimination of DON among different animal species [2]. Other factors that can be involved in differences in the severity of DON-related responses and susceptibility are age and sex [6,7]. Young pigs fed contaminated diets with DON experienced a greater reduction in weight gain compared with older animals [7]. This may be associated with a reduced capacity for DON metabolism and detoxification in the liver and gastrointestinal tract in younger animals [7]. A meta-analytical study of mycotoxins in pigs fed DON-contaminated diets showed that male pigs were more sensitive than females when growth performance was evaluated [7]. DON-related adverse effects vary from acute symptoms, such as emesis, diarrhea, and abdominal pain, to subacute and chronic effects, including reduced feed intake and body weight (BW) [2]. Immunotoxic, neurotoxic, and reprotoxic effects of DON are also of particular concern [3]. Most of these effects have been shown to be dose-dependent. Although poor growth and severe health issues have been reported at relatively high levels of DON contamination, the exposure of animals to low and moderate levels can result in cytotoxic effects that consequently lead to impairment of health and performance [8]. However, feeding weanling piglets with naturally contaminated diets with very low levels of DON (up to 840 µg/kg feed) had no effects on immune response, growth performance and biochemical parameters [9]. Evidence for direct effects on the fetus, including embryo-fetal toxicity, fetal malformation, and developmental disorders following maternal DON exposure, indicates a substantial risk for vertical DON transfer [10,11]. Understanding DON exposure during prenatal development and in early life has received considerable attention since the late 2000s, emphasizing the need for knowledge about vertical transmission of DON. Placental transfer of DON has been observed both in vitro using cell lines and in ex vivo studies [12,13]. Only a few in vivo studies investigated the transfer of DON across the placenta in pigs. Following feeding pregnant sows at mid-gestation (day 35–70) with diets containing 4.42 mg DON/kg feed, DON passed the placental barrier and was detected in fetal physiological samples, including urine, bile, serum, liver, and kidney [14]. In another experiment, the exposure of pregnant sows in the last third of gestation (day 75–110) with diets containing 9.57 mg DON/kg feed resulted in noticeable DON concentrations in urine, bile, and serum of the developing fetus, and serum concentrations of DON in piglets and sows were nearly similar [15]. It has been suggested that specificities in the placental structures in different species, as well as the time of exposure during gestation, may influence the extent of transplacental DON transfer [15].

Previous studies on DON transfer from dams to progeny have mainly focused on the placental transfer of DON. We are, however, not aware of any previous study on DON transfer from sow plasma into sow milk and the resulting lactational transfer to newborn piglets. In contrast, there are some reports on the transfer of feed-borne mycotoxins and their metabolites into the milk of dairy cattle [16,17]. Feeding dairy cows with contaminated diets containing 8.21 mg DON/kg resulted in very low levels of DON in the milk due to the rapid biotransformation of DON to de-epoxy DON (DOM-1), a less toxic metabolite, in the bovine rumen [17]. The microbial breakdown of trichothecenes in the rumen leads to a more significant lower DON uptake in ruminants than in pigs and other monogastric species. Consequently, higher DON plasma levels in pigs may contribute to a higher transfer rate into sow milk compared with cow milk [2].

Against this background, the goal of this study was to examine DON uptake in sows fed naturally contaminated feed during late gestation and lactation, and the transfer of DON from sows to fetuses/new-born piglets and suckling piglets. Additionally, the transfer rates of DON from sow plasma to the sow milk were determined.

## 2. Results

### 2.1. DON Uptake in Sows

#### 2.1.1. DON and DON Derivatives in Sow Feed

DON was detected at concentrations of 1.4 and 1.7 mg/kg in the DON level 1 and DON level 2 diets, respectively. The levels of 3-*O*-acetyl-DON (3-ac-DON) were 0.14 and 0.17 mg/kg in the DON level 1 and DON levels 2 diets, respectively. DON-3-*O*-β-d-glucoside (DON-3-Glc) was detected at concentrations of 0.32 and 0.36 mg/kg in the DON level 1 and DON level 2 diets, respectively. The levels of 15-*O*-acetyl-DON (15-ac-DON) were below the limits of detection LOD (0.05 mg/kg) in both DON-contaminated groups. The concentrations of DON, DON-acetates, and DON-3-Glc were all below the LOD (0.2 mg/kg for DON, 0.01 mg/kg for 3-ac-DON, 0.05 mg/kg for 15-ac-DON, and 0.03 mg/kg for DON-3-Glc) in the control feed.

The approximate average DON doses (μg/kg BW/day) in the groups fed experimental diets were calculated for the gestation and lactation periods, considering the sow mean feed intake and weights including gain during the study (Table 1). The estimated doses were not constant throughout the study, due to the changes in BW and feed intake during gestation and lactation. Notably, the feed consumption of sows is considerably higher during lactation compared to late gestation [18].

#### 2.1.2. DON and DON Metabolites in Sow Plasma

**Figure 1:**
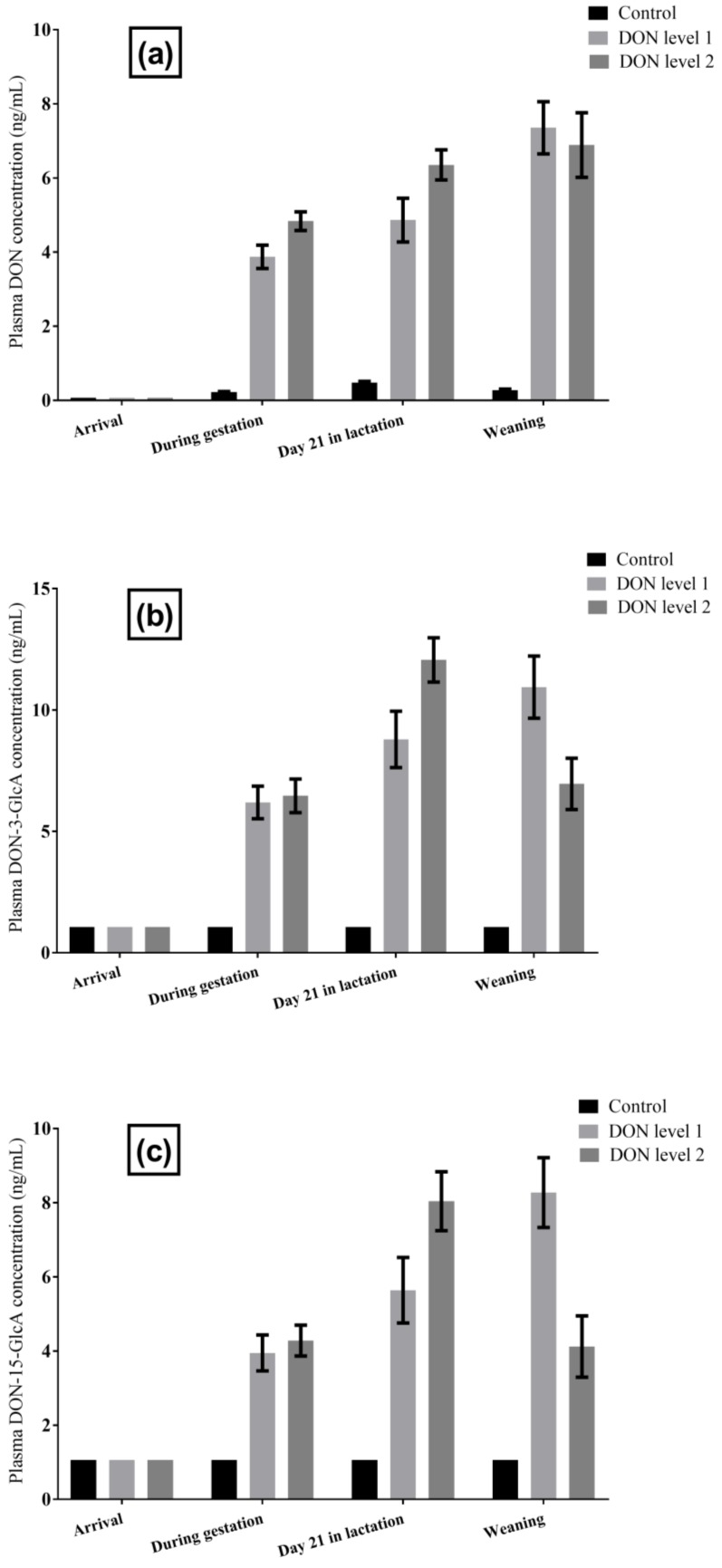
Effect of the experimental diets on plasma concentrations of (**a**) DON, (**b**) DON-3-GlcA, and (**c**) DON-15-GlcA in sows (*n* = 44) over the course of the exposure study. Error bars indicate the standard error of the mean (SEM).

The measured plasma concentrations of DON, DON-3-*O*-β-d-glucuronide (DON-3-GlcA), and DON-15-*O*-β-d-glucuronide (DON-15-GlcA) of the 44 sows sampled at different time points during the study are presented in Figure 1. In the samples taken at arrival in the farrowing unit, DON concentrations were below 0.1 ng/mL in all sows. During the experimental period, the mean DON plasma levels gradually increased in both groups fed with contaminated diets. The same was true for the levels of both DON-glucuronides in the DON level 1 group, whereas in the DON level 2 group, the mean DON-glucuronide levels decreased noticeably from day 21 in lactation until weaning (Figure 1). The plasma concentrations of DOM-1 were below the LOD in all sows at all sampling points.

The mean plasma concentrations of DON and DON glucuronides in the combined DON level 1 and 2 groups are shown in Table S1. The sow glucuronidation rates were calculated as the sum of plasma DON-3-GlcA + DON-15-GlcA concentrations divided by total DON (sum of plasma DON + DON-3-GlcA + DON-15-GlcA concentrations). The mean glucuronidation rates were 69%, 75%, and 67% at 10 days after arrival, on day 21 in the lactation, and at weaning, respectively.

The transfer rate of DON from the diet to sow plasma was calculated as plasma/diet ratio (plasma DON concentration divided by the DON concentration in feed). The resulting median plasma/diet ratios of the DON level 1 and DON level 2 groups were 0.003 and 0.003 at 10 days after arrival, 0.004 and 0.004 on day 21 in the lactation period, and 0.004 and 0.003 at weaning, respectively.

### 2.2. DON Transfer from Sows to Piglets

#### 2.2.1. DON Plasma Concentrations in Sows in the Transfer Study

DON was detected in almost all plasma samples from the sows included in the transfer study and fed with DON-contaminated diets (Table 2 and Figure S1a). In the control group, plasma DON concentrations higher than the LOD were measured at several time points in some sows (Table 2). The highest DON plasma concentrations were found in the sows fed DON level 1 diet at weaning (Table 2). During the lactation period, the mean DON concentrations in the two exposure groups were increasing toward weaning and concentrations at weaning were approximately five times higher than concentrations at farrowing.

DON-3-GlcA and DON-15-GlcA were detected in almost all plasma samples from the sows fed with DON-contaminated diets (Figure S1b,c). In the control group, plasma concentrations of DON glucuronides were below the LOD at all time points. The plasma concentrations of DON glucuronides increased from farrowing to weaning. The highest concentrations of DON glucuronides were found in sows fed DON level 1 diets at weaning. DOM-1 was not detected in any sow plasma sample.

#### 2.2.2. Piglet Survival and Growth Performance

In total, 75 newborn piglets were included, born to the 15 sows included in the transfer study (four from the control group, six from DON level 1 group, and five from DON level 2 group). Nine piglets died during the experiment: Three piglets died within 24 ± 8 h after parturition, four died between day 1 and day 7 after parturition, one died between day 7 and day 21, and one piglet died shortly before weaning. The data on the effects of DON-contaminated diets on piglet weight during the experimental period are shown in Table 3. There was a statistically significant effect of time on piglet weight, *F* (3, 191) = 1366, *p* < 0.0001. However, the interaction between time and treatments (DON) did not have a statistically significant effect on this variable *F* (6, 191) = 1.4, *p* = 0.22. No sex-dependent differences were found in the piglet BW.

#### 2.2.3. DON and DON Metabolites in Piglet Plasma

DON was detected in all plasma samples from piglets born and nursed by sows fed with DON-contaminated diets (Table 2 and Figure S2). In the control group, plasma DON concentrations close to the LOD were measured at several time points in some piglets. The highest DON plasma concentrations were found in the DON level 1 and 2 groups at birth (Table 2). During the lactation period, the mean DON concentrations in the two exposure groups decreased by 52% from birth to 12–36 h after parturition, by 89% until day 7 and by 74% until day 21. At weaning, the DON plasma concentrations were again slightly higher and were 43% lower compared with newborns.

The plasma concentrations of the metabolites DON-3-GlcA and DON-15-GlcA were below the LOD at most sampling points. At weaning, DON glucuronides were detected in the plasma of a few piglets at low levels. DOM-1 was not detected in any piglet plasma sample.

#### 2.2.4. Association between Plasma DON Concentrations in Sows and Piglets

**Figure 2:**
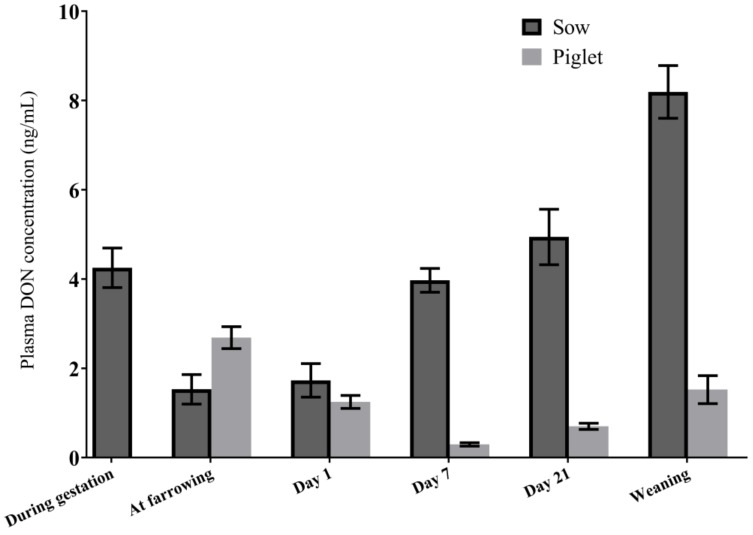
Plasma concentrations of DON in sows and piglets (combined DON level 1 and 2 groups) at birth, in the lactation period, and at weaning. The data represent means of *n* = 11 sows, and piglets (at farrowing: *n* = 55; day 1: *n* = 53; day 7: *n* = 49; day 21: *n* = 49; weaning: *n* = 48). Day 1 refers to sampling within 12–36 h after parturition. Error bars indicate standard error of the mean (SEM).

Plasma DON concentrations of the selected sows and their piglets in the transfer study are summarized in Table 2 and in the supplementary information (Figure S1a for the sows). Figure 2 shows plasma DON concentrations in the selected sows and their offspring combined for both DON exposure levels. The relationships between plasma samples of the sows and the piglets are expressed as the ratio between the concentrations of DON in piglet and sow plasma samples [14,15], and designated “piglet/sow plasma ratios” (Table 2). The piglet/sow plasma ratio refers to the mean of plasma DON concentrations in each litter of selected piglets, divided by that of their mothers. The ratios combined for both DON exposure levels were 2.14 at birth and 2.30 within 12–36 h after parturition (Table 4). From then on, the transfer ratios significantly reduced, with values of 0.08, 0.16, and 0.20 on day 7, day 21, and at weaning, respectively (Table 4).

The transfer of DON from the sows into their offspring is also illustrated by direct correlation between both DON plasma concentrations. DON plasma concentrations in piglets at day 1 postpartum and toward weaning may reflect several sources of intake (see Section 3). Therefore, correlations were determined for the respective plasma concentrations at birth as an indicator for placental transfer, and on day 7, during lactation as an indicator for lactational transfer. The plasma DON concentrations showed strong and significant sow-piglet correlation at birth (*r* = 0.88, *p* > 0.001). However, there was no association between plasma DON concentrations in sows and piglets on day 7.

#### 2.2.5. DON and DON Metabolites in Sow Milk

The repeated collection of milk samples allowed us to study the concentrations of DON over the course of lactation (Table 5). We used immunoaffinity columns for the solid-phase extraction of milk samples, which also retained DOM-1 and DON-15-GlcA, but not DON-3-GlcA. DON and DON-15-GlcA were detected in most (100% and 63%, respectively) of the milk samples collected from the sows in the transfer study, whereas DOM-1 was detected in 11% of the milk samples. The DON concentrations in the milk of DON-exposed sows increased consistently with about 55–60% from farrowing until weaning for both DON level groups (Table 5). DON-15-GlcA milk concentrations showed a comparable, but not so uniform, increase. The results show considerable inter-individual variation.

#### 2.2.6. DON Transfer from Sow Plasma to Milk (Lactational Transfer)

Lactational transfer was evaluated by calculating the sow milk/plasma (M/P) ratio at different time points during the lactation period, in accordance to Fleishaker [19]: M/P ratio = DON concentration in sow milk divided by DON plasma concentration in the sow.

**Figure 3:**
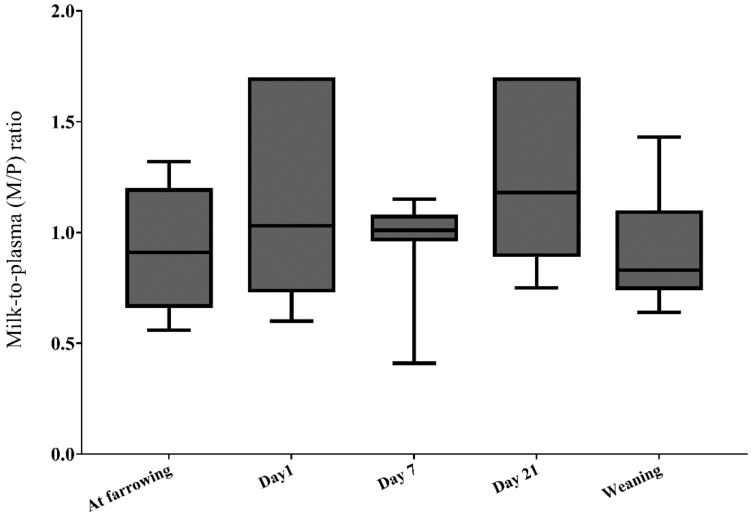
Milk/plasma (M/P) ratios at different stages in the lactation period for both DON level groups combined. Day 1 refers to assessment within 12–36 h after parturition.

M/P ratios were calculated for both DON level groups combined and at all sampling points. They varied from 0.41 to 1.7, indicating high inter-individual variability (Figure 3). The differences in M/P ratios between samples collected at birth (0.92 ± 0.28), within 12–36 h after parturition (1.11 ± 0.41), on days 7 (0.94 ± 0.26) and 21 (1.21 ± 0.36) in lactation, and at weaning (0.90 ± 0.24) were not statistically significant (*p* = 0.24).

The transfer of DON from sow plasma to sow milk was also evaluated by correlation analyses using the quantitative data for each sow (Figure S3a). DON concentrations in sow plasma and milk were correlated throughout the lactation period, at birth (*r* = 0.87, *p* < 0.001), within 12–36 h after parturition (*r* = 0.84, *p* < 0.01), on day 7 (*r* = 0.74, *p* < 0.01), on day 21 (*r* = 0.63, *p* < 0.05), and at weaning (r = 0.59, *p* = 0.08). Additionally, we looked for a possible correlation between DON concentrations in sow milk and in the plasma of the respective piglets (mean value) (Figure S3b). There was no strong correlation between DON in milk and DON in piglet plasma, neither on day 7 (*r* = 0.25, *p* = 0.46) nor at weaning (*r* = 0.42, *p* = 0.20), and DON in milk and DON in piglet plasma was not associated within 12–36 h after parturition and on day 21.

## 3. Discussion

Studies on the placental and lactational transport of DON are important in order to acquire knowledge of fetal and early life exposure. In the present in vivo study, we demonstrated that DON and the metabolites DON-3-GlcA and DON-15-GlcA were present in the plasma of sows that had been exposed to naturally DON-contaminated diets in the last stage of gestation and during lactation. We proved transfer of DON from exposed sows to their newborn piglets across the placenta and subsequently through milk. The data showed considerable variation among individuals and at the different sampling points in the lactation period, underlining that uptake and vertical transmission of contaminants is a complex mechanism. Additionally, the individual differences in sow feeding patterns may have affected DON concentrations in plasma and milk. The milk composition may also vary depending on demands made by the litter, the time of day, and the nutritional status of the sow.

### 3.1. DON Uptake in Sows

DON, DON-3-GlcA, and DON-15-GlcA were detected in dose-dependent plasma concentrations in sows in the lactation period, but not at weaning, where plasma concentrations for DON and DON metabolites were lower for DON level 2 than for DON level 1. The incongruence for DON at weaning might have resulted from increased variation between sows at this time point (DON < LOD for one sow). This explanation is, however, not applicable for DON-3-GlcA and DON-15-GlcA, for which the concentration differences between both dose levels were significant. The differences might also result from observed changes in the feed intake and BW during the study, which led to higher DON doses during lactation compared to late gestation. Placenta and uterus grow extensively in late gestation and contribute, together with the unborn piglets, to the higher BW that, in combination with decreased appetite, decreased the actual DON dose [20]. In contrast, the sow energy and nutrients requirements during lactation far exceed requirements in any other phase of the production cycle, and the higher feed intake, together with a lower BW after farrowing, increased the DON doses in this phase [21].

In the present study, the transfer of DON into plasma of sows (plasma/diet ratio) increased from arrival to weaning, where the ratio reached up to 0.008 in the DON level 1 group. The increasing DON uptake in sows during the lactation period was reflected in the observed rise in the plasma/diet ratio. Accordingly, the median plasma/diet ratio for both DON level groups during the whole study was 0.004, which is higher than that observed for sows fed a *Fusarium*-contaminated diet containing 4.42 mg DON/kg for 35 days in mid-gestation (serum/diet ratio 0.002) [14] and that reported in sows (0.001) after consuming a *Fusarium* toxin-contaminated diet of 9.57 mg DON/kg for 35 days in the last third of gestation [15]. In fattening pigs exposed to a *Fusarium* toxin-contaminated diet containing 6.68 mg DON/kg for 12 weeks, the mean serum/diet ratio was 0.002 [22]. Taken together, the pigs’ age, race, and the different stages in production cycle could all contribute to the observed differences in DON uptake. Comparing the uptake of DON in the current study with results from our recent study in growing pigs [23] leads to some interesting results. Calculated over the whole experiment, the DON plasma/diet ratios in the low DON (0.9 mg/kg feed) and medium DON (2.2 mg/kg) groups in the growing pig study were both close to 0.004, which is quite similar to the average value in the present study. This would indicate that age does not strongly affect DON uptake in pigs. However, if average plasma DON concentrations in the two studies are compared to average DON dose in µg/kg BW/day, the ratio between plasma concentrations and DON dose is about twice as high in the sows than in the growing pigs. Adult sows and young, growing pigs may not be directly comparable, but these results still indicate that the DON uptake in sows in late gestation and lactation appears to be similar to that in younger pigs.

### 3.2. Plasma Profiles of DON and DON-Glucuronides in Different Age Groups in Pigs

The present study allowed the comparison of plasma profiles of DON and DON-glucuronides in pigs of different ages after DON exposure via the diet. Using the data for sows and their offspring, as well as data from a previous study on growing pigs [23], we found that DON-3-GlcA is the main metabolite in pigs of all ages. Plasma concentrations of this metabolite were higher than those for DON and DON-15-GlcA after DON exposure in feed. In newborn piglets, measuring of DON and its metabolites in blood plasma immediately postpartum and before they received colostrum showed the extent of the placental transfer in the last stage of gestation. At this stage, DON-glucuronides were not detected in piglet plasma, indicating impaired or reduced placental transfer of these hydrophilic metabolites compared to DON. In suckling piglets, measurable levels of DON-3-GlcA and DON-15-GlcA were only found at weaning, although the metabolite concentrations in sow milk were in the same range as those for DON. However, after oral uptake in the piglets, the DON-glucuronides are probably not absorbed in the gut and excreted [24], or enzymatically split so that free DON is taken up. Another possible explanation for this finding is that metabolic DON conjugation to DON-glucuronides appears to not be fully developed in newborn piglets.

The mean glucuronidation rate for sows in both dose groups was 71% (Table S1) compared to 63% in growing pigs [16], which appears to be in line with the observation that younger animals have a lower capacity for DON metabolism [7]. Notably, the lower glucuronidation rate in the growing pig study might, in theory, be explained by the higher DON exposure (up to 5 mg DON/kg) compared to the present study (up to 1.7 mg DON/kg). DON is known to have hepatotoxic effects at higher levels [25], which might affect the glucuronidation capacity. However, an increase in serum hepatic enzymes, as an indication of hepatic damage, was not detected in the growing pig study [23]. We are not aware of any previous studies that evaluated age-related glucuronidation rates in DON exposed pigs. However, one previous study suggested that young mice are more susceptible than adult mice to DON-related effects due to higher plasma DON concentrations in weanling mice compared to adults, given identical doses of DON [26]. At 120 min after DON administration, DON plasma concentrations in young mice were close to the levels in adults, suggesting that age-related differences might be more related to DON uptake than to clearance [26].

### 3.3. Growth Performance of Piglets of DON-Exposed Sows

Adverse effects of feeding gestating and lactating sows with DON-contaminated diets on their piglets might be indirectly related to DON-induced reduction in feed intake of the exposed sows [18], and directly to placental or lactational transfer of this toxin to piglets. Therefore, both these aspects should be considered when evaluating the results from the present study. Average BW and daily weight gain of piglets in DON level 1 and DON level 2 were not significantly different from the control piglets. To the best of our knowledge, there are no previous studies that have evaluated individual BW development of piglets of sows exposed to DON during lactation. However, comparing effects on litter weight gain, the results of the current study concur with similar previous studies [27,28,29], but are in contrast with a report by Jakovac-Strajn et al. [30], who found that litter weight gain in sows receiving 5 mg DON/kg was significantly lower than the control. DON had no sex-related effects on piglet BW in the current study. This finding is in agreement with some previous studies, where no sex differences were observed in piglets of sows fed a *Fusarium* toxin-contaminated diet containing 9.52 mg DON/kg in the last third of gestation [15] or in the growing piglets receiving contaminated diets with up to 5 mg DON/kg for 42 days [23]. In contrast, male pigs have been shown to be more sensitive to DON-related toxicity than females in a previous meta-analytic study [13], in feeding experiments on fattening pigs [31], and in a study of fetuses of sows in mid-gestation [14].

### 3.4. DON Transfer from Sows to Unborn Piglets

Placental transfer of DON was investigated in vitro using human cell lines and ex vivo using human placenta [12]. The results from the in vitro study suggested that DON transferred slowly from the maternal side to the fetal side, and the ex vivo study showed that 21% of the initially incubated DON was transferred to the fetus [12]. In pigs, placental DON transfer was studied in vivo in pregnant sows in mid-gestation [14] and during late gestation [15]. The high DON levels in piglet plasma at birth in the present study indicated an efficient transfer of DON across the placenta in the last stage of pregnancy. There was a statistically significant association (*r* = 0.88, *p* < 0.001) in plasma DON concentrations between the sows and their newborns, comparable to the linear association observed in the reference study (*r*^2^ = 0.69) [15]. The mean and median piglet/sow plasma ratio at birth (mean: 2.14, *n* = 72; median: 1.80) was considerably higher than the median piglet/sow serum ratio (0.75, *n* = 91) reported for DON-exposed sows in the last third of gestation [15]. A transfer ratio close to unity would mean that DON absorbed by sows is present in the circulation of the fetuses at almost the same concentration as in the sows [15]. The differences in transfer ratios might result from the significantly reduced feed intake during farrowing in the present study, whereas in the previous experiment [15], the sows were euthanized in late gestation and before start of the stressful farrowing period. Since the plasma half-life (*t*½) of DON in pigs is relatively short (diet *t*½ 2–6 h) [5], plasma concentrations in the sows may have decreased considerably during the farrowing period. The piglets selected for the transfer study were consistently the firstborn, and plasma was sampled immediately at birth, whereas the samples from the corresponding sows were often collected after finished farrowing the whole litter. As an estimate for plasma DON concentrations in sows at the start of farrowing, we used the DON plasma concentrations in sows at 10 days after arrival/during gestation and calculated an expected plasma DON concentration just before farrowing, considering the reduction in feed allowance from approximately two days prior to expected farrowing. Using this estimate, piglet/sow plasma ratio was near unity, which was close to that of the referenced study [15].

### 3.5. DON Transfer from Sows to Suckling Piglets

The DON concentrations measured in colostrum and milk and the calculated M/P ratios indicated an efficient transfer of DON from sow plasma into milk, and showed no significant differences in transfer efficiency at different stages of lactation, since the M/P ratios were almost the same throughout the lactation period. The observed higher DON plasma concentrations in one-day-old piglets compared to later in lactation may indicate a better uptake of DON in the gut of piglets in the colostrum period than at a later stage. Some DON present in piglet plasma 12–36 h after birth could be a remnant of placental transfer, which would indicate a slower DON metabolism in newborns than in older animals. The gradual increase in DON in piglet plasma toward weaning may reflect that some piglets started to eat sow feed during late lactation. Due to practical limitations, it was not possible to prevent this in the present study.

To the best of our knowledge, the plasma-to-milk transfer of mycotoxins in different stages of lactation (i.e., colostrum versus mature milk) was previously investigated only in an ochratoxin A (OTA) exposure study in humans, where the OTA in colostrum was 2.5 times higher than in transient and mature milk [32]. These findings can, however, not be compared due to species differences and chemical differences between OTA and DON. Protein content is much higher in colostrum than in mature milk, and OTA is known for its protein-binding potential [33]. We are not aware of any studies that have investigated the binding of DON to milk proteins in pigs.

A limitation of our study was that neither the blood samples from sows and piglets nor the milk samples were collected at fixed time points during the day due to necessary adjustments to the course of farrowings and other practical limitations. However, the results from a previous study on growing pigs [23], where plasma DON concentrations were recorded at three times a day (8:00 a.m., 11:00 a.m., and 4:00 p.m.) during 35 days of exposure showed that the concentrations of DON and DON metabolites were relatively constant throughout the day, probably because of the ad libitum feeding. Therefore, we also assumed that the DON plasma concentrations of the sows were relatively stable throughout the day in the present study, except during farrowing and the first 12–36 h after parturition, when the sows had a temporarily reduced feed intake [18].

## 4. Conclusions

DON is transported efficiently across the placenta from sows to their piglets during the last stage of gestation. The transfer of DON from the lactating sows to suckling piglets via colostrum and milk was less efficient, particularly in mid-lactation. The differences in DON exposure of piglets at different times during lactation were apparently related to changes in DON uptake and metabolism in the piglets and not to changes in the transfer of DON from sow plasma to sow milk. The study results suggest that piglets of sows receiving DON-contaminated diets are at greater risk of DON exposure during late gestation and the first days after birth than during mid to late lactation.

## 5. Materials and Methods

### 5.1. Animals, Housing, and Management

**Figure 4:**
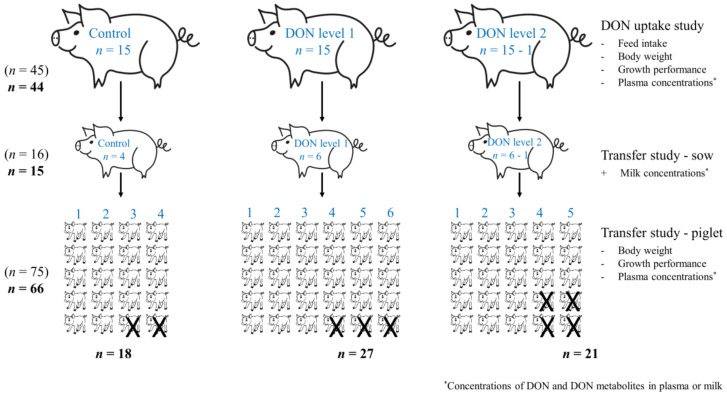
Flow chart of study design and animal groups in the DON uptake and transfer study.

This study was performed on 45 Norwegian Landrace × Yorkshire sows in a field trial during late gestation and lactation, using diets that were naturally contaminated with DON. It was conducted in a high-yielding specific pathogen free (SPF) unit from December 2015 to February 2016 [18]. The sows were divided into three groups (Figure 4): (1) control (DON < 0.2 mg/kg) (*n* = 15); (2) DON level 1 (1.4 mg DON/kg) (*n* = 15); and (3) DON level 2 (1.7 mg DON/kg) (*n* = 15). They were offered a restricted quantity of feed during late gestation, with a maximal allowance of 4 kg/day for each sow in this period. From approximately 2 days before expected farrowing, the daily feed allowance was reduced and adjusted to a daily feed allowance of between 2 and 3 kg per sow. After farrowing, the amount of feed offered to each sow was gradually increased, and the feeding automates had been adjusted to satisfy the requirements for a modified ad libitum feeding method. Further details of feeding and husbandry procedures were provided by Sayyari et al. [18]. All 45 sows in the field trial were included in the DON uptake study. However, one sow in the DON level 2 group and its piglets were later excluded from all calculations, because the plasma results of DON indicated a mistake in the feeding procedure for this sow; see comments under statistical analysis (Figure 4).

In the farrowing period, four sows from the control group, six sows from the DON level 1 group and six sows from DON level 2 group (one later excluded: see above) were selected for the transfer studies. The selection was performed randomly, but sows that started farrowing during the night were excluded. The farrowing of the selected sows was monitored continuously in order to take plasma samples from the newborn piglets before they received colostrum from the sows. The first five live-born and healthy piglets from each of the sows were tagged with plastic ear tags, COMBI 2000^®^ (OS ID^®^, Os, Norway), and sex was recorded. Each piglet were weighed within 48 ± 12 h after parturition, on days 7 and 21 in the lactation period, and at weaning, using a digital scale with 10-g accuracy according to the manufacturer (Premium 8006 GR-ST, EKS international SAS, Wittisheim, France).

This study was conducted according to the Norwegian regulations for animal testing (FOR-2015-06-18-761), which comply with EU Directive 2010/63/EU. A detailed description of the experiment, in accordance with the ARRIVE (Animal Research: Reporting of In Vivo Experiments) guidelines for reports of animal experiments, was evaluated and confirmed approvable by the Norwegian Animal Research Authority after completion (reference number: 2016/142986, Date: 18 August 2016).

### 5.2. Origin and Preparation of the Naturally Contaminated Experimental Diets

The DON-contaminated oats used for the production of the experimental diets for this study were harvested in Southern Sweden in 2013 and provided by Lantmännen, Stockholm, Sweden, and was identical to that used in our study on growing pigs [23]. Table S2 shows the analytical results for a wide range of mycotoxins, using a semi-quantitative multi-toxin screening method, at the Centre for Analytical Chemistry at Interuniversity Department for Agrobiotechnology (IFA) Tulln, Austria [34]. Detailed information on the composition of the experimental diets and feed was reported by Sayyari et al. [18].

### 5.3. Measuring of Feed Intake and Growth Performance of the Sows

In each farrowing pen, a plastic dispenser showed the quantity of feed consumed at each feeding to enable the simple recording of daily feed intake. In the current study, daily feed consumption refers to feed disappearance from the dispenser. This amount, however, was not adjusted for feed spillage or consumption of sow feed by piglets. Detailed information on registration of feed consumption and weighing the sows was given by Sayyari et al. [18].

### 5.4. Reagents for Chemical Analyses

Acetonitrile, methanol, and water for liquid chromatography high-resolution mass spectrometry (LC-HRMS) analysis were of Optima™ (Fisher Scientific, Fair Lawn, NJ, USA) LC-MS quality, whereas acetonitrile for sample preparation (gradient quality) was obtained from Romil (Cambridge, UK). Ammonium acetate and glacial acetic acid were of p.a. quality (Merck, Darmstadt, Germany). 4-deoxynivalenol (DON), DOM-1, 3-ac-DON, 15-ac-DON, and DON-3-Glc were purchased from Romer Labs (Tulln, Austria), whereas DON-3-GlcA and DON-15-GlcA were available from work performed in a previous study [35].

### 5.5. Analysis of Mycotoxins in Experimental Diets

Samples of 2.5 g from each of the experimental diets were milled with a Retsch ZM 100 mill (Retsch GmbH & Co., KG, Haan, Germany) and placed into 50-mL centrifuge tubes, and 10 mL acetonitrile/H_2_O/formic acid (80:19.9:0.1, *v*/*v*/*v*) was added. The mixture was vortexed for 30 s and extracted for 30 min using a horizontal shaker at 175 min^−1^. After centrifugation at 4000× *g* for 10 min (4 °C), the supernatants were transferred into new 50-mL tubes, and the remaining solid material was extracted with 10 mL of acetonitrile/H_2_O/formic acid (20:79.9:0.1, *v*/*v*/*v*) by shaking for an additional 30 min. The two extracts were combined and kept at 4 °C before final centrifugation (4000× *g*, 10 min, 4 °C). Finally, 0.5 mL aliquots of the combined supernatants were centrifuged for 1 min at 15,000× *g* through 0.22-μm nylon filters (Costar Spin-X, 0.22 µm; Corning, Inc., Corning, NY, USA). The prepared samples were analyzed for DON, DON-3-Glc, 3-ac-DON, and 15-ac-DON with a previously validated LC-HRMS method [36]. The LOD in feed matrix were 14 µg/kg for DON, 26 µg/kg for DON-3-Glc, 5.9 µg/kg for 3-ac-DON, and 52 µg/kg for 15-ac-DON. The prepared experimental diets contained DON as the main contaminant, whereas other mycotoxins that commonly occur in Norwegian cereal grain were below the LOD or detected at low levels (Table S2). The hay provided to the sows was not analyzed for mycotoxin content. However, all hay was of good quality with no signs of fungal contamination.

### 5.6. Blood and Milk Sampling for Analysis of Mycotoxins

#### 5.6.1. DON Uptake Study

Blood samples from all sows were taken from the milk vein (*v. subcutanea abdominis*) without any form of immobilization, while they were lying down or standing [37], using 9-mL lithium-heparin tubes. The samples were collected upon arrival in the farrowing unit, after 10 days (during gestation), on day 21 in lactation, and at weaning. Plasma was prepared by centrifugation at 1500× *g* for 10 min at room temperature, stored frozen in 2-mL cryogenic vials (Nalgene, Nalge Company, Rochester, NY, USA), and delivered to the Norwegian Veterinary Institute for analysis.

#### 5.6.2. Transfer Study

Blood samples of the sows included in the transfer study were taken from the milk vein (*v. subcutanea abdominis*), using 9-mL heparin tubes at farrowing, within 12–36 h after parturition (day 1), on days 7 and 21 in the lactation period, and at weaning. Blood samples from the respective piglets were collected from the jugular vein (*v. jugularis*), using 2.7-mL S-Monovette^®^ Lithium-Heparin tubes (Sarstedt AG & Co., KG, Nümbrecht, Germany) immediately after birth (before the newborns received colostrum), within 12–36 h after parturition, on days 7 and 21 in the lactation period, and at weaning.

Colostrum was collected shortly after the onset of parturition. Transient milk was sampled on day 1 after parturition and mature milk on days 7 and 21 in the lactation period, and at weaning. In this study, 2 mL to 5 mL milk was sampled from the first available teat into plastic vials by manual expression and without oxytocin administration, after the milk ejection was initiated by the suckling behavior of sows and piglets. The collected samples were stored frozen (−20 °C) in 5-mL cryogenic vials (Greiner Bio-One International GmbH, Monroe, NC, USA) until analyzed.

Neither the blood samples from sows and piglets nor the milk samples were collected at fixed times during the day. Sample collections were always started in the morning, but due to necessary adjustments to the course of farrowings and other practical limitations, sampling was sometimes not finished until late in the evening.

### 5.7. Sample Preparation of Plasma for Analysis of Mycotoxins

Plasma samples (250 µL) were transferred into conical 15-mL plastic tubes (Corning Inc., Corning, NY, USA), mixed with 750 µL acetonitrile, vortexed for 15 s, and sonicated (Branson 3200, Emerson, St. Louis, MO, USA) for 5 min. Proteins were precipitated by centrifugation at 2000× *g* for 10 min at 4 °C (Beckman Coulter, Brea, CA, USA), and supernatants were transferred to 10-mL conical glass tubes and evaporated to dryness at 60 °C using a gentle stream of nitrogen. Dried samples were stored refrigerated, re-dissolved in 200 µL water, vortexed for 15 s, sonicated for 5 min, and transferred to HPLC vials for LC-HRMS analysis. Each round of analyses included at least one blank pig plasma sample, which had been fortified with 5.3 or 26 ng/mL of DON, DON-3-GlcA, and DON-15-GlcA and 11 or 52 ng/mL DOM-1.

### 5.8. Sample Preparation of Sow Milk for Analysis of DON, DOM-1 and DON-15-GlcA

Milk samples were allowed to thaw at room temperature, transferred to 1.5-mL Eppendorf tubes, and then centrifuged at 20,000× *g* for 20 min. Positive control samples containing either 27.5 ng/mL of DON, 27.6 ng/mL of DOM-1, and 16.5 ng/mL of DON-15-GlcA, or 2.75 ng/mL of DON, 2.76 ng/mL of DOM-1, and 1.65 ng/mL of DON-15-GlcA were prepared by spiking blank colostrum or milk samples with 20 µL of the appropriate standard solution in water. Aliquots of the supernatants (400 µL) were transferred to immunoaffinity columns (ImmunoClean CF for DON, aokin AG, Berlin, Germany), which had been rinsed with 3 mL phosphate buffered saline (PBS). The immunoaffinity columns were designed for the clean-up of DON, but were previously shown to retain also DOM-1 and DON-15-GlcA, but not DON-3-GlcA [38]. Following application of the supernatants, they were washed with 5 mL PBS and dried under vacuum for approximately 30 s. The columns were then allowed to soak with 1.5 mL methanol for 2 min and eluted. The eluates were evaporated to dryness at 60 °C using a gentle stream of nitrogen. Residues were dissolved in 200 µL water, vortexed, and then sonicated for 5 min. The solutions were filtered through 0.22 µm Nylon membranes (Spin-X, Corning Inc., New York, NY, USA) and transferred to chromatography vials.

### 5.9. Quantitative Analysis of Mycotoxins in Plasma and Milk

The plasma samples were analyzed for DON, DOM-1, DON-3-GlcA, and DON-15-GlcA. Samples were chromatographed at 30 °C using a Vanquish ultra-high-performance liquid chromatography (UHPLC) (Thermo Fisher Scientific, Waltham, MA, USA) and a 100 × 2.1 mm i.d. Acquity ultra performance liquid chromatography (UPLC) HSS T3 column (1.8 μm; Waters, Milford, MA, USA) including a 5 × 2.1 mm i.d. XSelect HSS T3 VanGuard pre-column (2.5 µm, 100 Å, Waters, Milford, MA, USA). The flow rate of the mobile phase was 0.5 mL/min, and the injection volume was 6 μL. Eluent A was water and eluent B was 95% acetonitrile (both containing 5 mM ammonium acetate and 0.1% acetic acid). The column was eluted isocratically with 100% A for 1 min, and then the mobile phase composition was changed to 15% B using a linear gradient over 15 min. After flushing the column for 2.5 min with 100% B, the mobile phase composition was returned to the initial conditions, and the column was re-equilibrated for 2.9 min. The UHPLC-system was coupled to a Q-Exactive™ Hybrid Quadrupole-Orbitrap high-resolution mass spectrometer (Thermo Fisher Scientific, Waltham, MA, USA) equipped with a heated electrospray ion source (HESI-II). The HESI-II interface was operated at 300 °C in the negative ionisation mode, and the parameters were adjusted as follows: Spray voltage, 4 kV; capillary temperature, 250 °C; sheath gas flow rate, 35 units; auxiliary gas flow rate, 10 units; and S-lens radio frequency (RF) level, 55. The data were acquired in the selected ion monitoring (SIM)/data-dependent MS^2^ (dd-MS2) mode targeting the [M + acetate]^−^ ions for DON and DOM-1 (*m*/*z* 355.1387 and 339.1438, respectively) and the [M − H]^−^ ions for the DON-glucuronides (*m*/*z* 471.1497) with a quadrupole isolation width of 2 *m*/*z* and a mass resolution of 70.000 full width half-maximum (FWHM) at *m*/*z* 200 for SIM. The presence of a target ion above a threshold intensity of 5 × 10^3^ triggered a MS^2^ scan for analyte verification (dd-MS2) using a normalized collision energy of 35%. The mass resolution during dd-MS2 was set to 17.500 FWHM. The automatic gain control (AGC) target was set to 5 × 10^5^ ions including a maximum injection time (IT) of 250 ms during SIM, whereas for dd-MS2, the AGC target was 5 × 10^4^ and the IT was 200 ms.

Matrix-matched, 1/x weighed calibration curves were plotted for DON, DOM-1, DON-3-GlcA, and DON-15-GlcA using plasma of untreated pigs (blank). Xcalibur version 2.2 or 2.3 (Thermo Fisher Scientific, Waltham, MA, USA) was used for data processing. The overall spike recoveries were (standard deviations in parentheses) DON 80% (11), DOM-1 74% (12), DON-3-GlcA 67% (17), and DON-15-GlcA 65% (14). The LODs were 0.1 ng/mL for DON, 0.2 ng/mL for DOM-1, and 1.5 ng/mL for DON-3-GlcA and DON-15-GlcA.

Instrument calibration for quantification of DON, DOM-1, and DON-15-GlcA in sow milk was carried out using 1/x weighed calibration curves and water as solvent for standards. The overall spike recoveries were (standard deviations in parentheses) DON 54% (16), DOM-1 59% (8.5), and DON-15-GlcA 119% (55) in colostrum, and DON 55% (18), DOM-1 58% (8.7), and DON-15-GlcA 89% (26) in milk. The LODs were estimated to 0.2 ng/mL for DON and DOM-1, and to 0.7 ng/mL for DON-15-GlcA.

### 5.10. Statistical Analysis

The analyses of DON concentrations in sow plasma indicated that a mistake in the feeding of one sow in the DON level 2 group had occurred. The plasma concentrations of DON and DON metabolites in this sow were below the LOD in 5 of 8 assessments. Therefore, this sow and its piglets were excluded from all calculations and statistical evaluations. Thus, the final dataset included 44 sows in the DON uptake study (15 controls, 15 at DON level 1, and 14 at DON level 2); and 15 sows (4 controls, 6 at DON level 1, and 5 at DON level 2) in the transfer study.

Piglet growth performance and plasma concentration data were analyzed by a mixed model in JMP^®^, Version 10 (SAS Institute Inc., Cary, NC, USA). The level of significance was set to 0.05 in all models, and results with *p*-values between 0.05 and 0.1 were considered significant trends. If not otherwise specified, all results are expressed as the mean ± standard deviation (SD). The normality of distribution of the different parameters was controlled by residual and predicted values plot, normal-percentile plots, and the Shapiro-Wilk test. If the *p*-value in the Shapiro-Wilk test was greater than 0.05, the data were considered normally distributed. Data that were not normally distributed were analyzed by non-parametric Wilcoxon’s rank sum test. Levene’s test was used to check the assumption of homogeneity of variances. If the *p*-value of Levene’s test was greater than 0.05, variances were considered equal. If the output generated from the application of one-way Analysis of Variance (ANOVA) indicated significant differences, the post-hoc Tukey-Kramer Honestly Significant Difference (HSD) test was used for multiple comparisons and the identification of significant differences (*p* < 0.05). Interactions that were not statistically significant were removed from the models by backward elimination.

DON concentrations measured in plasma samples from the sows fed experimental diets and in selected piglets (pooled per sow) were compared using the non-parametric Wilcoxon Each Pair test (*p* < 0.05) as these data were not normally distributed.

Correlations between DON concentrations in the plasma of sows and their respective piglets were analyzed by a non-parametric Spearman’s rank-order correlation test for non-normally distributed data and by Pearson correlation test for normally distributed data (*r_s_* for Spearman’s rank-order and *r* for Pearson correlation test: *p* < 0.05). In order to analyze the correlation between plasma DON concentrations in sows and piglets, we merged the data from both groups fed DON-contaminated feed (DON level 1 and DON level 2) into a single data set for piglets and sows. Wilcoxon’s rank sum test (*p* < 0.05) was used for statistical comparisons of M/P ratios. In the statistical calculations, concentrations below the LOD were represented by the LOD divided by the square root of 2 [39].

During the data evaluations and statistical analyses, missing values and outliers were encountered. Therefore, imputation analysis for missing values and winsorization was used whenever appropriate [40]. Results from the control groups were not included in the calculation and presentation of ratios and correlations, because the low values in the control groups may create false impressions of these variables.

## Figures and Tables

**Table 1:** Daily DON doses in the sows during late gestation and lactation.

Diets	Parameters	Late Gestation	Lactation
		Mean ± SD ^a^	Mean ± SD
Control (*n* = 15)	Body Weight ^b^	290 ± 40	251 ± 42
	ADFI ^c^	3.8 ± 0.2	6.6 ± 0.7
	DON intake ^d^	<0.8	<1.3
	DON dose ^e^	<2.6	<5.4
DON level 1 (*n* = 15)	Body Weight	301 ± 34	258 ± 40
	ADFI	3.4 ± 0.3	6.5 ± 1.2
	DON intake	4.7 ± 0.4	9.1 ± 1.7
	DON dose	15.7 ± 1.5	35.4 ± 3.3
DON level 2 (*n* = 14)	Body Weight	303 ± 25	255 ± 34
	ADFI	3.7 ± 0.3	6.1 ± 0.4
	DON intake	6.2 ± 0.5	10.3 ± 0.9
	DON dose	20.6 ± 1.9	40.9 ± 5.0

^a^ Standard deviation; ^b^ Mean body weight (kg); ^c^ Average daily feed intake (kg/day); ^d^ Daily intake of DON in feed (mg/day): ADFI × DON conc. in feed; ^e^ Daily DON dose (μg/kg BW/day).

**Table 2:** Plasma concentrations of DON and ratios between matched sows (*n* = 15) ^a^ and piglets (*n* = 75) ^a^ plasma over the course of experiment.

Sampling Point	10 days after Arrival			At Farrowing			Day 1			Day 7			Day 21			Weaning		
Diets	Con	Level 1	Level 2	Con	Level 1	Level 2	Con	Level 1	Level 2	Con	Level 1	Level 2	Con	Level 1	Level 2	Con	Level 1	Level 2
**Sow plasma (ng/mL)**																		
Mean ± SD	0.22 ± 0.04	3.71 ± 1.81	4.90 ± 0.57	0.10 ± 0.04	1.79 ± 1.12	1.13 ± 0.90	<LOD	2.65 ± 0.70	0.81 ± 0.76	0.24 ± 0.05	4.22 ± 1.14	3.67 ± 0.37	0.46 ± 0.05	4.31 ± 2.65	5.70 ± 0.69	0.30 ± 0.11	9.06 ± 1.57	7.15 ± 1.99
Median	0.22	3.98	4.79	0.09	1.77	0.96	<LOD	2.88	0.53	0.23	3.96	3.56	0.46	4.52	6.02	0.27	9.42	7.27
Range	0.17–0.25	0.48–5.52	4.36–5.66	<LOD–0.16	0.68–3.71	0.23–2.38	<LOD	1.67–3.50	<LOD–1.69	0.18–0.30	3.03–6.06	3.20–4.16	0.41–0.53	0.52–7.82	4.80–6.44	0.20–0.45	7.2–11.4	5.0–10.1
**Piglet plasma (ng/mL)**																		
Mean ± SD				0.47 ± 0.28	2.97 ± 0.85	2.35 ± 0.65	0.14 ± 0.12	0.94 ± 1.11	1.62 ± 0.20	<LOD	0.26 ± 0.13	0.32 ± 0.18	0.12 ± 0.11	0.80 ± 0.43	0.58 ± 0.16	0.11 ± 0.09	1.69 ± 1.39	1.20 ± 1.41
Median				0.41	2.69	2.26	<LOD	0.73	1.61	<LOD	0.25	0.32	<LOD	0.71	0.51	<LOD	1.05	0.61
Range				<LOD–1.61	1.74–4.74	1.34–3.43	<LOD–0.37	0.49–6.66	1.20–2.08	<LOD	<LOD–0.57	<LOD–0.79	<LOD–0.38	0.45–2.70	0.38–0.94	<LOD–0.41	0.32–5.27	0.20–6.33
**Piglet/sow plasma ratio ^b^**																		
Mean ± SD				–	2.05 ± 0.87	3.64 ± 3.25	–	0.30 ± 0.08	6.78 ± 8.41	–	0.06 ± 0.03	0.09 ± 0.03	–	0.20 ± 0.09	0.11 ± 0.04	–	0.21 ± 0.17	0.20 ± 0.19

^a^*n* = 4 control sows, *n* = 6 sows fed with DON level 1, *n* = 5 sows fed with DON level 2; *n* = 75 live-born piglets; day 1: *n* = 72; day 7: *n* = 68; day 21: *n* = 67; weaning: *n* = 66. ^b^ Missing values and outliers are treated. –: Piglet/sow plasma ratio was not calculated for control.

**Table 3:** Effect of diet on piglet growth performance.

Parameters	*n*	Diets		
		Control ^a^	DON Level 1 ^a^	DON Level 2 ^a^
Piglet body weight (kg):				
48 ± 12 h after parturition	71	2.2 ± 0.4	1.8 ± 0.3	1.8 ± 0.4
Day 7	68	3.1 ± 0.8	2.7 ± 0.5	2.9 ± 0.8
Day 21	67	7.4 ± 1.8	7.1 ± 1.2	7.2 ± 1.6
Weaning	66	11.9 ± 2.4	10.7 ± 1.6	11.0 ± 2.1
Average weaning age (days)		35	33	33
Total average daily gain (g/day)		297 ± 67	290 ± 48	292 ± 59
		(*n* = 18)	(*n* = 27)	(*n* = 21)

^a^ Mean ± SD.

**Table 4:** Piglet/sow plasma ratios for the combined results of the DON level 1 and DON level 2 groups ^a^.

Sampling Point	10 Days after Arrival	At Farrowing	Day 1	Day 7	Day 21	Weaning
**Sow plasma (ng/mL)**						
Mean ± SD	4.25 ± 1.47	1.53 ± 1.04	1.73 ± 1.19	3.97 ± 0.88	4.94 ± 2.06	8.19 ± 1.96
Median	4.61	1.24	1.68	3.73	5.56	7.80
Range	0.48–5.66	0.23–3.71	<LOD–3.50	3.03–6.06	0.52–7.82	5.0–11.4
**Piglet plasma (ng/mL)**						
Mean ± SD		2.69 ± 0.81	1.25 ± 0.89	0.29 ± 0.16	0.70 ± 0.35	1.48 ± 1.41
Median		2.55	1.11	0.30	0.64	0.86
Range		1.34–4.74	0.49–6.66	<LOD–0.79	0.38–2.70	0.20–6.33
**Piglet/sow plasma ratio ^b^**						
Mean ± SD		2.14 ± 0.80	2.30 ± 2.81	0.08 ± 0.03	0.16 ± 0.08	0.20 ± 0.17

^a^ Combined plasma concentrations in sows in the DON level 1 and DON level 2 groups (*n* = 11) and the matched piglets. The number of the piglets in each sampling point; *n* = 55 live-born piglets; day 1: *n* = 53; day 7: *n* = 49; day 21: *n* = 49; weaning: *n* = 48. ^b^ Missing values and outliers are treated.

**Table 5:** DON, DOM-1, and DON-15-GlcA concentrations in milk of sows included in the transfer study ^a^.

Sampling Point	At Farrowing			Day 1			Day 7			Day 21			Weaning		
Diets	Con	Level 1	Level 2	Con	Level 1	Level 2	Con	Level 1	Level 2	Con	Level 1	Level 2	Con	Level 1	Level 2
**DON (ng/mL)**															
Mean ± SD	0.42 ± 0.22	1.35 ± 0.52	1.20 ± 0.39	0.29 ± 0.09	2.14 ± 0.30	1.43 ± 1.49	0.48 ± 0.05	3.72 ± 1.98	4.14 ± 1.19	0.50 ± 0.00	5.67 ± 1.72	5.75 ± 1.52	0.73 ± 0.17	7.49 ± 1.99	7.03 ± 2.79
Range	0.25–0.75	0.82–2.29	0.81–1.65	0.23–0.43	1.68–2.44	0.46–4.06	0.43–0.53	1.33–5.97	3.08–6.20	0.50–0.50	2.97–7.09	4.61–8.29	0.57–0.97	5.4–10.5	4.1–10.4
**DOM-1 (ng/mL)**															
Mean ± SD	<LOD	<LOD	0.15 ± 0.02	<LOD	<LOD	<LOD	<LOD	<LOD	<LOD	<LOD	<LOD	0.18 ± 0.05	<LOD	0.17 ± 0.04	0.22 ± 0.06
Range	–	–	<LOD–0.19	–	–	–	–	–	–	–	–	<LOD–0.24	–	<LOD–0.23	<LOD–0.30
**DON-15-GlcA (ng/mL)**															
Mean ± SD	<LOD	3.18 ± 1.42	4.39 ± 0.77	<LOD	2.42 ± 1.57	2.17 ± 0.86	<LOD	1.89 ± 1.12	3.52 ± 3.95	<LOD	2.62 ± 1.04	7.00 ± 4.55	0.82 ± 0.27	6.37 ± 4.65	10.8 ± 1.9
Range	–	1.86–5.02	3.49–5.14	–	0.66–4.37	1.20–3.08	–	<LOD–3.51	1.20–10.5	–	1.75–4.51	2.3–13.2	0.57–1.06	2.6–13.1	8.6–13.6

^a^*n* = 4 control sows, *n* = 6 sows fed with DON level 1, *n* = 5 sows fed with DON level 2.

## Supplementary Materials

The following are available online at http://www.mdpi.com/2072-6651/10/12/517/s1; Figure S1: Effect of experimental diets on plasma concentrations of (a) DON, (b) DON-3-GlcA, and (c) DON-15-GlcA in sows included in the transfer study; Figure S2: Effect of experimental diets on plasma DON concentrations in piglets over the course of study; Figure S3: The relationship between DON concentrations in milk samples and plasma concentrations of DON in (a) sows and (b) piglets regardless of treatment (for DON level 1 and DON levels 2 groups) over the course of study; Table S1: Sow plasma concentrations of DON and DON-glucuronides, and glucuronidation rates determined over the course of the experiment; Table S2: Toxin contents in the oats used for the production of the experimental diets, as measured with multi-toxin liquid chromatography-tandem mass spectrometry (LC-MS/MS) at the Centre for Analytical Chemistry at IFA Tulln, Austria.
