# Supplementary material for: Transfer of Deoxynivalenol (DON) through Placenta, Colostrum and Milk from Sows to Their Offspring during Late Gestation and Lactation
Source: Toxins (Basel). 2018 Dec 4;10(12):517. doi: 10.3390/toxins10120517 (PMC6315731; doi:10.3390/toxins10120517)
Supplement: Supplementary file 1 [file toxins-10-00517-s001.pdf]

Supplementary Materials: Transfer of Deoxynivalenol (DON) through Placenta, Colostrum and Milk from Sows to Their Offspring during Late Gestation and Lactation

Amin Sayyari, Silvio Uhlig, Christiane Kruse Fæste, Tore Framstad and Tore Sivertsen

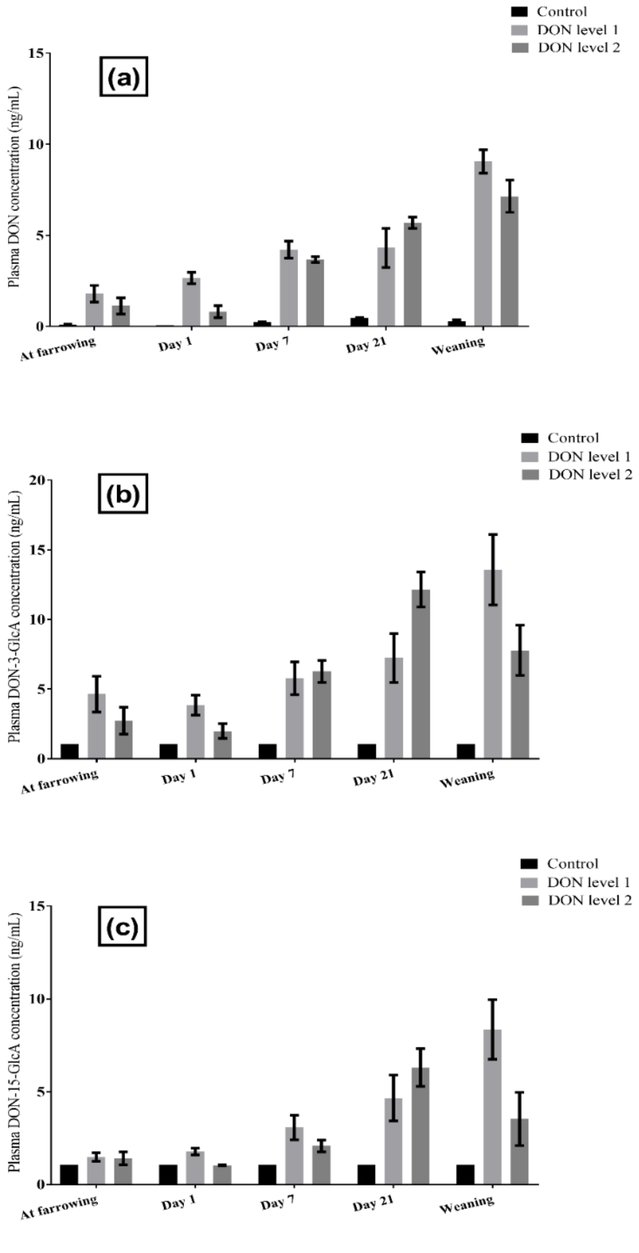

Figure S1. Effect of experimental diets on plasma concentrations of (**a**) DON, (**b**) DON-3-GlcA and (**c**) DON-15-GlcA in sows included in the transfer study. Day 1 refers to 12–36 h after parturition. Error bars are the standard error of the mean (SEM).

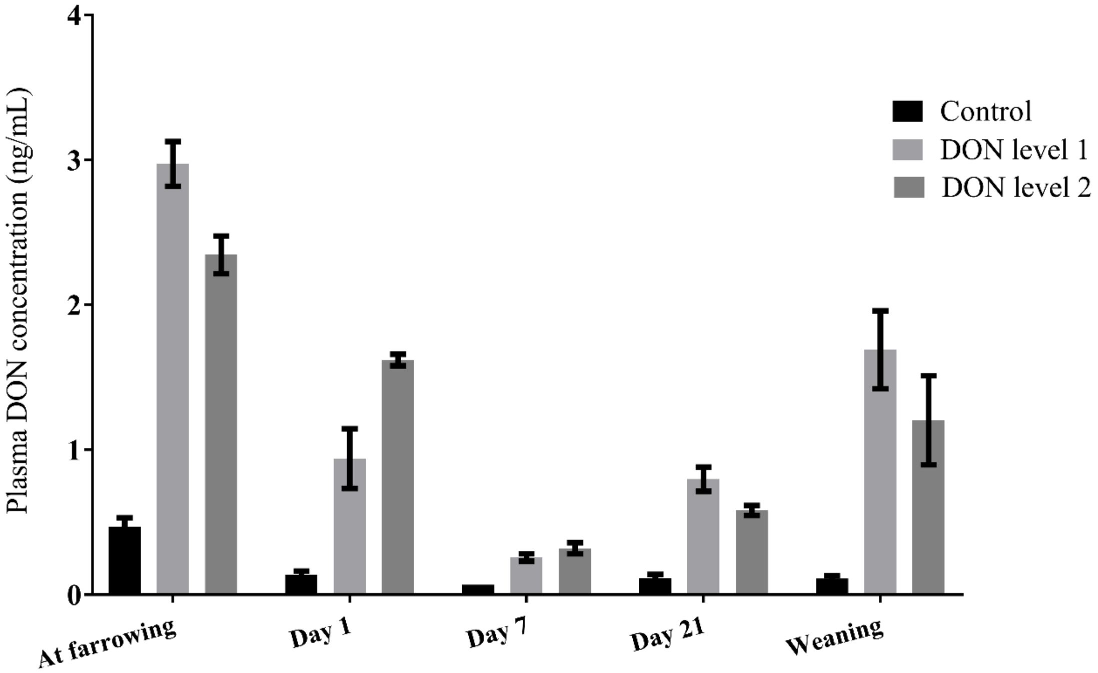

Figure S2. Effect of experimental diets on plasma DON concentrations in piglets over the course of study. Day 1 refers to the assessment within 12–36 h after parturition. Error bars are the standard error of the mean (SEM).

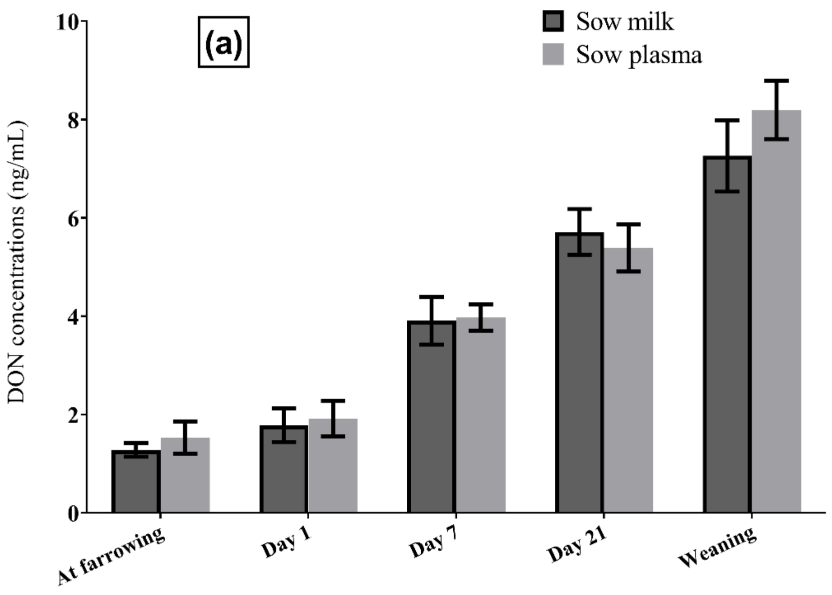

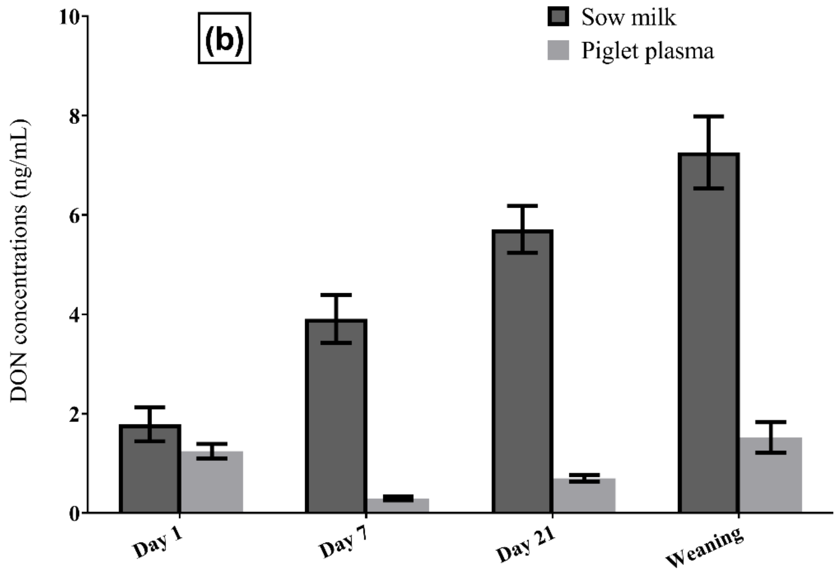

Figure S3. The relationship between the DON concentrations in milk samples and plasma concentrations of DON in (**a**) sows and (**b**) piglets regardless of treatments (for DON level 1 and DON levels 2 groups) over the course of study. Each column is the average of DON levels in milk and plasma DON concentrations of all individuals in sow or piglet groups. Day 1 refers to within 12–36 h after parturition. Error bars are the standard error of the mean (SEM).

Table S1. Sow plasma concentrations of DON and DON–glucuronides, and glucuronidation rates determined over the course of the experiment [a].

Sampling Point	DON (ng/mL)	DON-3-GlcA (ng/mL)	DON-15-GlcA (ng/mL)	Σ total DON (ng/mL)	Glucuronidation Rate (%)
Arrival	< LOD	< LOD	< LOD	< LOD	–
10 days after arrival	4.34 ± 1.18	6.31 ± 2.55	4.11 ± 1.71	14.77 ± 4.83	69
Day 21 in lactation	5.58 ± 2.06	10.37 ± 4.28	6.80 ± 3.38	22.75 ± 9.06	75
Weaning	7.13 ± 2.96	9.02 ± 4.87	6.27 ± 3.97	22.42 ± 9.57	67
Average [b]	5.68 ± 1.22	8.57 ± 2.18	5.72 ± 2.02	19.98 ± 4.33	71

[a] The concentrations presents the average of sow plasma concentrations in two contaminated groups, DON level 1 and DON level 2; [b] The values are calculated based on the average of plasma concentrations at the three sampling points for the combined DON level 1 and 2 groups sows in the DON uptake study, excluding the value at arrival.

Table S2. Toxin contents in the oats used for the production of the experimental diets, as measured with multi-toxin liquid chromatography-tandem mass spectrometry (LC–MS/MS) at the Centre for Analytical Chemistry at IFA Tulln, Austria.

Toxins (µg/kg)	Oats [a]	
	Sample 1	Sample 2
15-Hydroxyculmorin	499	500
15-Hydroxyculmoron	182	193
5-Hydroxyculmorin	387	441
Alternariol	15.3	2.12
Alternariolmethylether	1.58	0.64
Andrastin A	< LOD	< LOD
Apicidin	54.5	49.5
Ascochlorin	6.37	0.98
Asperfuran	< LOD	< LOD
Asperglaucide	5.38	3.91
Asperphenamate	12.08	9.51
Aspterric acid	< LOD	< LOD
Aurofusarin	1771	1310
Beauvericin	13.3	14.4
Brevianamid F	10.4	9.15
Butenolid	19.6	21.9
Chanoclavin	0.24	0.27
Chrysogin	134	144
Citreorosein	5.42	5.83
Culmorin	2139	1996
Curvularin	14.4	7.52
cyclo(L-Pro-L-Tyr)	79.2	63.9
cyclo(L-Pro-L-Val)	95.5	75.9
Deoxynivalenol	3788	3975
Diplodiatoxin	< LOD	< LOD
DON-3-glucoside	843	869
Elymoclavine	< LOD	< LOD
Emodin	4.86	5.70
Enniatin A	0.39	0.36

Enniatin A1	2.83	3.09
Enniatin B	14.9	14.7
Enniatin B1	13.0	13.2
Enniatin B2	0.69	0.81
Enniatin B3	0.00	0.00
Epiequisetin	0.85	0.91
Equisetin	1.88	1.16
Ergometrine	< LOD	10.44
Ergometrinine	0.35	0.82
Fallacinol	< LOD	< LOD
Fellutanine A	15.5	11.9
Fonsecin	< LOD	< LOD
Fumonisin B1	< LOD	< LOD
Fumonisin B2	< LOD	< LOD
Fumonisin B3	< LOD	< LOD
Fusaric acid	< LOD	< LOD
Fusarinolic acid	< LOQ [b]	< LOQ
HT-2 toxin	9.82	< LOD
Infectopyron	175	189
Iso-Rhodoptilometrin	< LOD	0.30
Kojic acid	< LOD	< LOD
Macrosporin	< LOD	< LOD
Moniliformin	9.46	28.3
Mycophenolic acid	< LOD	< LOD
N-Benzoyl-Phenylalanine	5.07	3.48
Neoechinulin A	22.6	33.1
Nivalenol	402	399
Ochratoxin A	< LOD	< LOD
Pestalotin	< LOD	< LOD
Physcion	< LOD	< LOD
Questiomycin A	< LOD	< LOD
Quinolactacin A	0.14	0.10
Rugulusovin	5.00	3.58
Siccanol	< LOQ	< LOQ
T-2 toxin	10.1	< LOD
Tentoxin	1.24	2.08
Tenuazonic acid	82.6	40.2
Tryptophol	111	85.3
Xanthotoxin	< LOD	< LOD
Zearalenone	38.1	58.5

[a] The samples 1 and 2 are the replicates of the same batch of oats. [b] Limit of Quantitation (LOQ).
